# “Spotting” *Mycobacterium bovis* infection in leopards (*Panthera pardus*) – novel application of diagnostic tools

**DOI:** 10.3389/fimmu.2023.1216262

**Published:** 2023-09-01

**Authors:** Rachiel Gumbo, Wynand J. Goosen, Peter E. Buss, Lin-Mari de Klerk-Lorist, Konstantin Lyashchenko, Robin M. Warren, Paul D. van Helden, Michele A. Miller, Tanya J. Kerr

**Affiliations:** ^1^ DSI-NRF Centre of Excellence for Biomedical Tuberculosis Research, SAMRC Centre for Tuberculosis Research, Division of Molecular Biology and Human Genetics, Faculty of Medicine and Health Sciences, Stellenbosch University, Cape Town, South Africa; ^2^ South African National Parks, Veterinary Wildlife Services, Kruger National Park, Skukuza, South Africa; ^3^ Skukuza State Veterinary Office, Department of Agriculture, Land Reform and Rural Development, Skukuza, South Africa; ^4^ Chembio Diagnostic Systems, Inc., Medford, New York, NY, United States

**Keywords:** DPP^®^ Vet TB assay, gene expression assay, GeneXpert^®^ MTB/RIF Ultra qPCR assay, interferon-gamma release assay, leopard, *Mycobacterium bovis*, *Panthera pardus*, tuberculin skin test

## Abstract

**Background:**

*Mycobacterium bovis* (*M. bovis*) is the causative agent of animal tuberculosis (TB) which poses a threat to many of South Africa’s most iconic wildlife species, including leopards (*Panthera pardus*). Due to limited tests for wildlife, the development of accurate ante-mortem tests for TB diagnosis in African big cat populations is urgently required. The aim of this study was to evaluate currently available immunological assays for their ability to detect *M. bovis* infection in leopards.

**Methods:**

Leopard whole blood (n=19) was stimulated using the QuantiFERON Gold Plus In-Tube System (QFT) to evaluate cytokine gene expression and protein production, along with serological assays. The GeneXpert^®^ MTB/RIF Ultra (GXU^®^) qPCR assay, mycobacterial culture, and speciation by genomic regions of difference PCR, was used to confirm *M. bovis* infection in leopards.

**Results:**

*Mycobacterium bovis* infection was confirmed in six leopards and individuals that were tuberculin skin test (TST) negative were used for comparison. The GXU^®^ assay was positive using all available tissue homogenates (n=5) from *M. bovis* culture positive animals. *Mycobacterium bovis* culture-confirmed leopards had greater antigen-specific responses, in the QFT interferon gamma release assay, *CXCL9* and *CXCL10* gene expression assays, compared to TST-negative individuals. One *M. bovis* culture-confirmed leopard had detectable antibodies using the DPP^®^ Vet TB assay.

**Conclusion:**

Preliminary results demonstrated that immunoassays and TST may be potential tools to identify *M. bovis*-infected leopards. The GXU^®^ assay provided rapid direct detection of infected leopards. Further studies should aim to improve TB diagnosis in wild felids, which will facilitate disease surveillance and screening.

## 1 Introduction

Leopards (*Panthera pardus*) occupy diverse habitats across Africa and Asia, but are listed as vulnerable, with some populations decreasing (1). These iconic species play a role in terrestrial ecosystem functions, biodiversity maintenance, ecotourism industries, and cultural rituals in some South African societies (2, 3). However, apex predators including leopards are threatened by ecological disturbances, climate change, and infectious diseases (4, 5). Infections in wild felids include multi-host pathogens such as *Mycobacterium bovis* (*M. bovis*), a member of the *Mycobacterium tuberculosis* complex (MTBC), which is acquired by ingesting infected prey (4). This results in animal tuberculosis (TB), a chronic progressive disease in domestic animals and wildlife, as well as zoonotic TB in humans (6, 7).

Conservation programs often require translocation of individuals to maintain genetic diversity or reintroduce animals into past or new regions (8). However, the presence of infectious disease could threaten the success of these programs (9). Since movement of infected felids carries a potential risk of introducing *M. bovis* into naive populations, it is crucial to develop accurate ante-mortem tests to diagnose *M. bovis* infection prior to translocation as well as perform surveillance. However, ante-mortem TB diagnosis and management have been hampered by the lack of reliable validated tests for many wildlife species (10, 11).

Currently, the tuberculin skin test (TST) is the most widely used ante-mortem test for diagnosing *M. bovis* infection in large wild felids (11). However, performing two immobilizations 72 hours apart is considered impractical in free-ranging wildlife. Therefore, there is an urgent need for the development of single-capture TB diagnostic assays. Although serological tests have been evaluated for TB detection in African lions (*Panthera leo*), results suggested insufficient sensitivity of the assay during early infection (12). However, assays based on *in vitro* cell-mediated immune (CMI) responses appear to be more sensitive for identifying infected individuals (11). Stimulation with specific mycobacterial peptides, such as the early secretory antigenic target 6 kDa (ESAT-6) and culture filtrate protein 10 kDa (CFP-10) in the QuantiFERON^®^-TB Gold Plus (QFT) tubes (Qiagen, Hilden, 40724, Germany), has been used to elicit cytokine/chemokine responses, measured by gene expression or enzyme-linked immunosorbent assays (ELISAs). This approach has been explored in a variety of wild carnivore species including cheetahs (*Acinonyx jubatus*), spotted hyenas (*Crocuta crocuta*), African wild dogs (*Lycaon pictus*), and African lions (13–16). Previous studies have shown the upregulation of the C-X-C motif ligand 9 (*CXCL9*) gene to be a sensitive diagnostic biomarker for *M. bovis* infection in African lions and a single cheetah (16, 17). Cytokine release assays (CRA) have also shown diagnostic potential in cheetahs and African lions (13, 18). Therefore, the aim of this study was to evaluate immunological assays, previously validated in African lions and cheetahs, to identify potential TB diagnostic tests for leopards. The development of a blood-based test would facilitate screening of individuals and disease surveillance of leopard populations.

## 2 Materials and methods

Between 2011 and 2022, blood (n = 19) and post-mortem tissue (n = 6) samples were opportunistically collected for purposes unrelated to this study from free-ranging leopards in the Greater Kruger National Park (GKNP), South Africa, which is considered endemic for *M. bovis* (6). Blood samples were collected during the first immobilization for those animals that were immobilized twice to perform the TST. The sex and age category (adult > 4 years; sub-adult 2-4 years; juvenile <2 years) were recorded at the time of sample collection. Wildlife veterinarians performed examinations and leopards in good body condition, without visual evidence of illness or injury were released after immobilization. Leopards that were in poor body condition, with significant wounds, injuries, or other clinical abnormalities (ex. impaled with porcupine quills) and assessed to have a poor long-term prognosis were euthanized. Post-mortem examinations were performed, and tissues were collected for *M. bovis* detection. Testing for other infections or diseases was not performed. Since samples were acquired opportunistically, not all samples were available from every individual. South African Veterinary Council (SAVC)-registered wildlife veterinarians were responsible for all animal-related procedures. The study protocol was approved by the Stellenbosch University Animal Care and Use Research Ethics Committee (SU-ACU-2020-14571) and the Stellenbosch University Biological and Environmental Safety Research Ethics Committee (SU-BEE-2021-22561). Section 20 approval was granted by the South African Department of Agriculture, Land Reform and Rural Development (DALRRD 12/11/1/7/2A-1143NC).

Post-mortem samples, including lymph nodes (head, thoracic, abdominal, peripheral) and lungs, were collected from six leopards and processed for mycobacterial culture as previously described (19). Bacterial isolates were genetically speciated by genomic regions of difference PCR (20). As a rapid ancillary method to mycobacterial culture, the GeneXpert^®^ MTB/RIF Ultra (GXU^®^) qPCR assay (Cepheid Inc., Sunnyvale, CA 94089, USA) was used to confirm the presence of MTBC DNA directly in tissue homogenates as previously described (19). A positive MTBC result was defined as all readouts except “MTBC not detected” (21).

Once immobilized, the single intradermal cervical test (SICT) and single intradermal comparative cervical test (SICCT) were performed by veterinarians in accordance with the procedures described for lions (22). Individuals were immobilized 72 hours later, and skin thickness (ST) measured at the bovine and avian purified protein derivative (PPD) injection sites and observed for oedema, redness, and necrosis. Results were calculated and categorized as previously described in lions (23). In this study, the single intradermal comparative cervical test (SICCT) results were used to classify animals as TST positive or negative; an animal was considered SICCT positive if an increase in skin thickness at the bovine PPD site was ≥ 2 mm and the bovine PPD response was greater than the avian PPD response (23).

Whole blood (lithium heparin and serum) was collected in BD Vacutainer^®^ blood collection tubes (Becton, Dickinson and Company, Sparks, MD 21152, USA) prior to performing the TST. Blood was transported in a Styrofoam box to the laboratory at room temperature and processed less than two hours after collection. Blood was processed for serum and stimulated using QFT, as previously described (13, 16). Pokeweed mitogen (PWM; 10 µg/ml final concentration in phosphate buffered saline (PBS); Sigma-Aldrich, St. Louis, MO 63118, USA) was added to the QFT mitogen tube to ensure adequate stimulation. After 24 hours of incubation at 37°C for 24 hours, plasma supernatant was collected and stored at -80°C for CRAs, while the remaining cell pellet was stabilized in 1.3 ml of RNALater^®^ (Ambion, Austin, TX, 78744, USA) and stored at -80°C for cytokine gene expression assays (GEA).

Following the manufacturer’s instructions, sera were screened using the Chembio DPP^®^ Vet TB for Elephants rapid serological assay (Chembio Diagnostic Systems, Inc., Medford, NY 11763, USA), as previously described for lions (12). This assay detects the presence of antibodies to mycobacterial antigens MPB83 (test line 1) and ESAT-6/CFP-10 (test line 2), using a species non-specific detection system. Quantitative results were obtained using a DPP^®^ optical reader (Chembio) to measure reflectance in relative light units (RLU), with a RLU ≥ 5 (manufacturer’s recommended visual cut-off value) considered antibody positive (12).

The concentration (ng/µl) and purity (A260/A280 and A260/A230 ratios) of extracted RNA from the QFT cell pellets were measured in single replicates per sample using a Nanodrop 1000 spectrophotometer (ThermoFisher Scientific, Wilmington, NC 28401, USA), reversed transcribed to cDNA, and was used to evaluate the performance of the cytokine GEAs, as previously described for use in African lions (16). To evaluate real-time quantitative polymerase chain reaction (qPCR) primer compatibility between lions and leopards, partial messenger RNA (mRNA) transcripts for the target genes *CXCL9*, C-X-C motif chemokine ligand 10 (*CXCL10)*, and interferon-gamma (*IFN-γ*), and reference genes including tyrosine 3-monooxygenase/tryptophan 5-monooxygenase activation protein zeta polypeptide (*YWHAZ*), TATA box-binding protein (*TBP*), β-2-microglobulin (*B2M*), and glyceraldehyde-3-phosphate dehydrogenase (*GAPDH*), were amplified and sequenced using lion PCR primers (sequencing primers), as previously described (16). The newly generated leopard sequences were authenticated (24) and deposited into the NCBI GenBank^®^ genetic sequence database (http://www.ncbi.nlm.nih.gov/genbank/) (25) under the accession numbers OP894012 – OP894028. The gene sequences of lions and leopards were aligned (26) to evaluate sequence identity between species as well as to evaluate compatibility of qPCR primers for downstream analysis.

Real-time qPCR amplification efficiencies for both target and reference genes were determined using a five-fold serial dilution over a 625-fold range using pooled cDNA from five randomly selected leopards. Inefficient genes (90% > efficiency > 110%) were excluded from further analysis. To validate the use of the relative quantification method described (27), the amplification efficiencies of the most stable reference gene real-time qPCRs were compared to those of the target genes to evaluate compatibility (28, 29), as described in African lions (16). Relative abundances of *CXCL9* and *CXCL10* mRNA and changes in regulation were determined as previously described (27, 30). The infection status of leopards based on *CXCL9* GEA results was determined using the previously calculated African lion cut-off value (5-fold change) (16).

The R&D Systems Feline DuoSet^®^ ELISA development kits (R&D Systems, Inc., Minneapolis, MN 55413, USA) for TNF-α (catalogue no. DY2586) and IL-1β (catalogue no. DY1796), and the Mabtech Cat IFN-γ ELISA^Basic^ kit (catalogue no. 3122-1H-20; Mabtech AB, Nacka Strand, SE-131 28, Sweden) were evaluated to determine if native leopard cytokines could be detected in stimulated whole blood plasma as previously shown in cheetah (13) and lions (18). A four-parameter logistic (4PL) regression analysis was performed using GraphPad Prism 7 for Windows (version 7.04, GraphPad Software, Inc., San Diego, CA 92108, USA; www.graphpad.com). The differences between unstimulated and mitogen responses for the three cytokine release assays were evaluated using a one-tailed paired Student t-test. The infection status of leopards based on QFT Mabtech Cat IGRA results was determined using the previously calculated African lion cut-off value (33 pg/ml) (18).

Proportions of GEA and IGRA test positive leopards in *M. bovis* culture positive and TST-negative cohorts were compared using the Fishers’ exact test. Blood-based assay results were also evaluated in parallel. Parallel test interpretation was performed by categorizing an individual leopard as positive if either test result was positive, based on previously described African lion cut-off values. A p-value < 0.05 was considered statistically significant. All statistical tests were performed using GraphPad Prism 7 for Windows (version 7.04, Graphpad Software, Inc.).

## 3 Results

In this study, a total of 19 leopards, 12 males and 7 females, including 16 adults, 1 sub-adult and 2 juveniles, were tested using different ante-mortem (TST, DPP^®^, GEA, and IGRA) and post-mortem (mycobacterial culture and GXU^®^) techniques. Results are summarized in **Table 1**. *Mycobacterium bovis* infection was confirmed in six leopards by mycobacterial culture of post-mortem tissues and speciation by RD PCR. Using tissue homogenates, available for five leopards, the GXU^®^ was successful in detecting MTBC DNA in all five *M. bovis* culture-confirmed leopards, providing same day results (**Table 1**).

**Table 1:** Summary of demographic information, *Mycobacterium bovis* infection status, and test results of 19 free-ranging leopards (*Panthera pardus*) sampled in Greater Kruger National Park, South Africa and tested using different ante-mortem (TST, DPP^®^, GEA, and IGRA) and post-mortem (mycobacterial culture and GXU^®^) techniques.

Leopard Identification Number	Age	Sex	Sample Year	TST	TB lesions present	Mycobacterial Culture and Speciation	GXU^®^	DPP^®^	QFT *CXCL9* GEA	QFT *CXCL10* GEA	QFT Mabtech Cat IGRA
KNP-19/260	Adult	Female	2019	pos	yes	*M. bovis*	MTBC detected - low	pos	pos	unknown	neg
KNP-19/07/01	Adult	Male	2019	pos	yes	*M. bovis*	MTBC detected - high	neg	neg	unknown	pos
KNP-19/279	Adult	Male	2019	pos	yes	*M. bovis*	MTBC detected - low	neg	pos	unknown	pos
KNP-18/660	Juvenile	Female	2018	n/d	no	*M. bovis*	n/d	neg	pos	unknown	pos
KNP-22/728	Adult	Male	2022	n/d	yes	*M. bovis*	MTBC detected - medium	neg	pos	unknown	neg
KNP-22/853	Adult	Male	2022	n/d	yes	*M. bovis*	MTBC detected - low	neg	neg	unknown	neg
KNP-11/121	Adult	Male	2011	neg	n/a	n/d	n/d	n/d	n/d	unknown	invalid
KNP-11/236	Adult	Male	2011	neg	n/a	n/d	n/d	n/d	n/d	unknown	neg
KNP-16/155	Adult	Female	2016	neg	n/a	n/d	n/d	neg	neg	unknown	invalid
KNP-18/426	Juvenile	Female	2018	neg	n/a	n/d	n/d	neg	neg	unknown	neg
KNP-20/58	Adult	Female	2020	neg	n/a	n/d	n/d	neg	neg	unknown	neg
KNP-14/228	Adult	Male	2014	n/d	n/a	n/d	n/d	n/d	pos	unknown	neg
KNP-17/682	Adult	Female	2017	n/d	n/a	n/d	n/d	neg	n/d	unknown	n/d
KNP-17/752	Adult	Male	2017	n/d	n/a	n/d	n/d	neg	pos	unknown	pos
KNP-18/35	Adult	Male	2018	n/d	n/a	n/d	n/d	neg	neg	unknown	neg
KNP-18/234	Adult	Female	2018	n/d	n/a	n/d	n/d	neg	neg	unknown	neg
KNP-18/526	Adult	Male	2018	n/d	n/a	n/d	n/d	neg	neg	unknown	neg
KNP-18/425	Sub-adult	Male	2018	n/d	n/a	n/d	n/d	neg	pos	unknown	pos
KNP-22/576	Adult	Male	2022	n/d	n/a	n/d	n/d	neg	n/d	unknown	pos

Age, Adult > 4 years; sub-adult 2-4 years; juvenile < 2 years; TST, tuberculin skin test; GXU^®^, GeneXpert^®^ MTB/RIF Ultra; MTBC, Mycobacterium tuberculosis complex; DPP^®^, Dual path platform^®^ Vet TB assay; QFT, QuantiFERON^®^-TB Gold Plus; GEA, gene expression assay; CXCL9, C-X-C motif ligand 9; CXCL10, C-X-C motif chemokine ligand 10; IGRA, interferon gamma release assay; M. bovis, Mycobacterium bovis; pos, positive; neg, negative; n/d, not done; n/a, not applicable; unknown, In the absence of an existing CXCL10 cut-off value, the GEA test results for CXCL10 were unknown.

Eight leopards were tested using the TST, including three *M. bovis* culture-confirmed individuals. Skin fold thickness measurements at the avian and bovine PPD injection sites are shown in **Supplementary Table S1**. The three culture positive leopards were SICCT positive, and the remaining animals were SICCT negative, even though measurement values for KNP-11/121 and KNP-11/236 were unavailable (**Table 1**). None of the TST negative (presumed *M. bovis*-uninfected) leopards were euthanized and therefore, there were no post-mortem tissues available for mycobacterial culture confirmation.

Sixteen leopards were screened for antibodies to mycobacterial antigens using the DPP^®^ Vet TB assay. Of the six culture-confirmed *M. bovis*-infected individuals, leopard KNP-19/260 was the only individual with detectable antibodies to mycobacterial antigen MPB83, and none to antigen ESAT6/CFP10 (**Table 1**, **Supplementary Table S2**). Five culture positive leopards had post-mortem lesions consistent with TB; only KNP-18/660, a juvenile leopard with confirmed *M. bovis* infection did not have gross TB lesions present. The remaining 10 leopards (3 TST-negative, 7 with unknown status) were seronegative.

Stimulated whole blood cell pellets were available from 15 leopards. Three mitogen-stimulated leopard samples were selected for initial PCRs using previously described lion primers (16). Partial mRNA transcripts for reference (*YWHAZ*, *TBP* and *GAPDH*), and target (*CXCL9, CXCL10*, and *IFN-γ*) genes were amplified, although the reference gene *B2M* PCR, failed to produce amplicons. Sequence alignments for the target and reference genes showed >95% sequence identity when lion and leopard sequences were compared. In addition, lion qPCR primers (16) showed 100% sequence identity to the leopard primer region, and successfully amplified these genes in leopard samples. Amplification efficiencies of all genes were 90-110% (**Supplementary Table S3**), except *IFN-γ* and *TBP*. Reference gene *YWHAZ* was confirmed to be the most stably expressed gene and was compatible with *CXCL9* and *CXCL10*.

**Figure 1:**
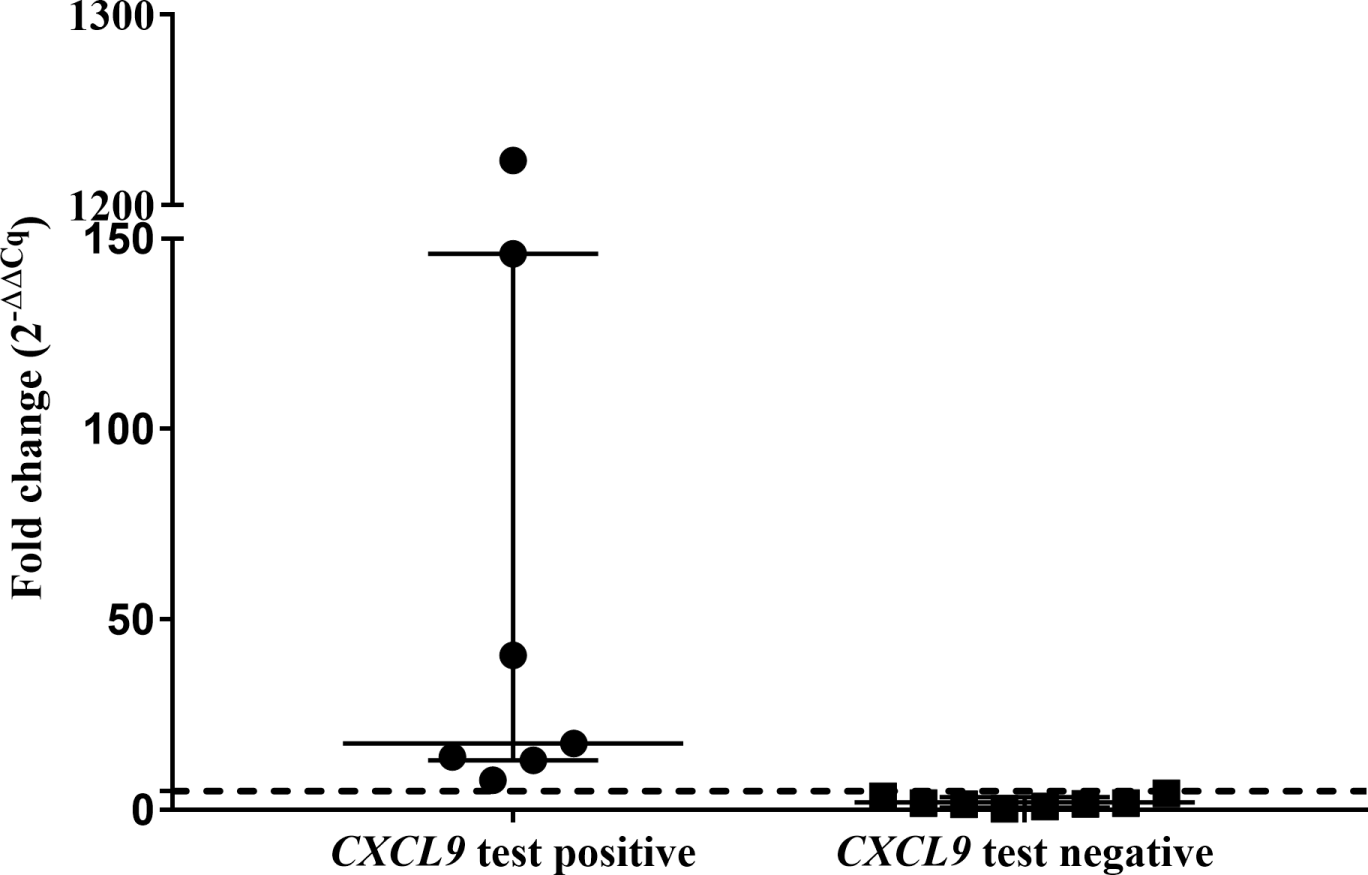
Antigen-specific *CXCL9* mRNA fold change (2^−ΔΔCq^) of test positive (n=7) and test negative leopards (n=8) using the QFT *CXCL9* GEA. Medians and inter-quartile ranges are indicated by horizontal bars. The lion assay cut-off value (5-fold change) is shown as a dotted line on the y-axis (16). There was a statistically significant difference between the test results (p = 0.0002).

The abundances of target genes *CXCL9* and *CXCL10* relative to the optimal reference gene are shown as fold change values in **Supplementary Table S4**. Poor mitogen responses (fold change <5) were observed in 8 of the 15 leopards. Using the African lion cut-off value (fold change ≥5), the *CXCL9* GEA correctly identified 4/6 *M. bovis* culture-confirmed leopards (**Figure 1**). Similar results were observed for the *CXCL10* GEA with higher upregulation observed for the same four leopards (**Supplementary Table S4**). Although KNP-22/853 was negative in the *CXCL9* GEA (4.42-fold change), upregulation of antigen-specific *CXCL10* (53.2-fold change) was observed. Leopards with TST-negative results had low level expression of both *CXCL9* and *CXCL10* (**Supplementary Table S4**). Of the six leopards with unknown infection status, three had positive *CXCL9* results along with *CXCL10* upregulation (**Supplementary Table S4**). When *CXCL9* GEA results for leopards that were *M. bovis* culture positive (n = 6) were compared to TST-negative animals (n = 3), no significant association was observed (p = 0.12).

All three feline cytokine ELISAs (TNF-α, IL-1β, and IFN-γ) were able to detect cytokine production in QFT mitogen stimulated leopard samples (**Supplementary Table S5**). Significant differences in cytokine concentrations were observed between QFT mitogen stimulated and QFT nil samples, using R&D Feline IL-1β (p = 0.009), R&D Feline TNF-α (p = 0.046) and Mabtech cat IFN-γ (p = 0.046, **Supplementary Table S5**) ELISAs. The coefficients of variation (CV) for the TNF-α and IFN-γ release assays were below 30% while the IL-1β assay had an increased CV (173.3%) because of the background signal observed at the 1:2 sample dilution (**Supplementary Table S5**).

A subset of samples from *M. bovis* culture positive (n=3) and TST-negative leopards (n=1) produced valid mitogen responses in all three cytokine release assays using a 1:4 dilution of leopard plasma (**Supplementary Table S6**). The R&D Feline IL-1β and Mabtech Cat IFN-γ ELISAs detected an antigen-specific response in 2/3 *M. bovis* culture positive leopards, while the R&D Feline TNF-α ELISA showed an antigen-specific response in only 1/3 *M. bovis* culture positive leopards (**Supplementary Table S6**). Since the QFT Mabtech Cat IGRA has been validated for lions, it was selected for further evaluation.

**Figure 2:**
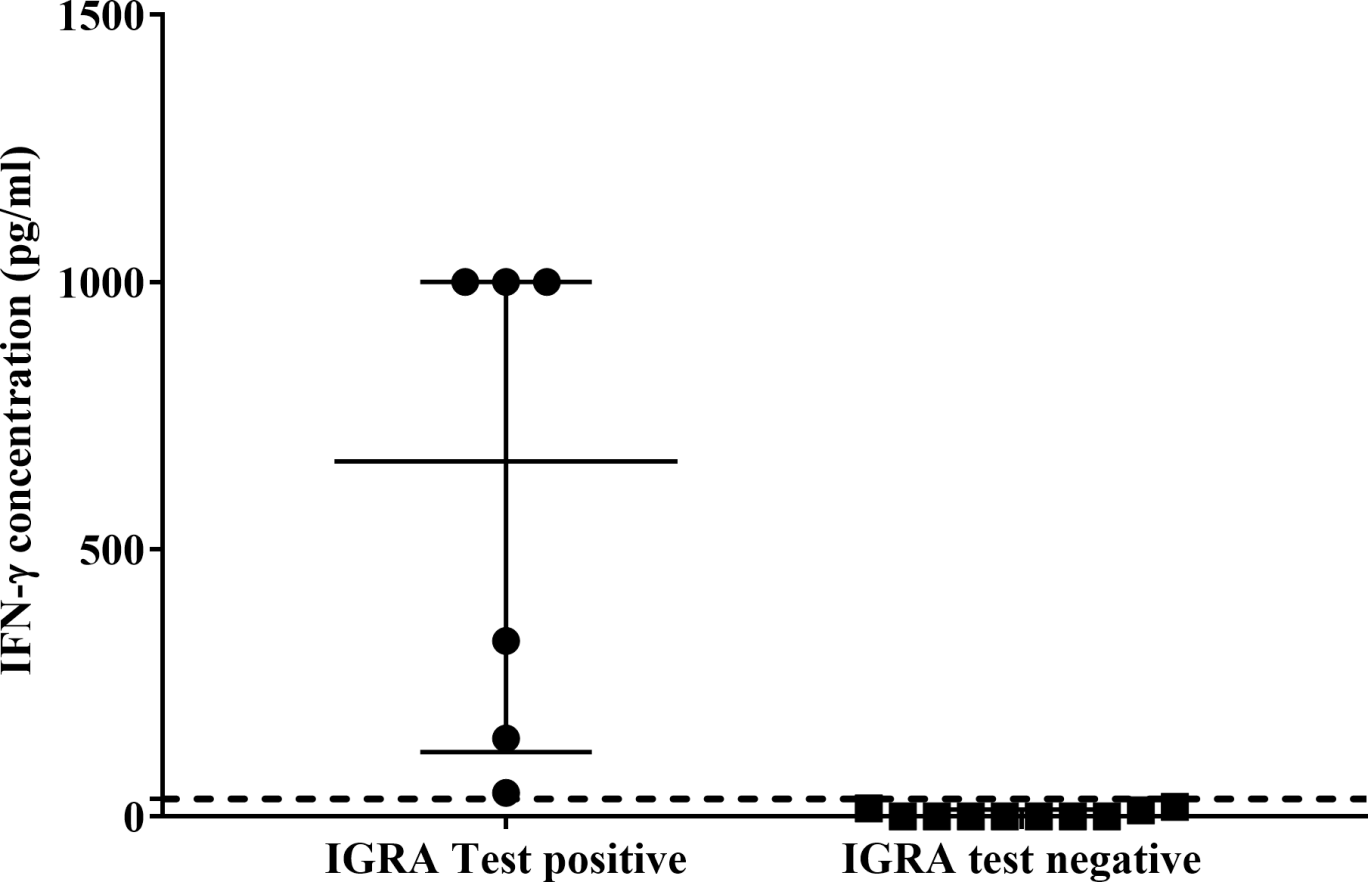
Antigen-specific interferon-gamma (IFN-γ) concentrations (pg/ml) of QFT Mabtech Cat IGRA positive (n=6) and negative (n=10) leopards. Medians and inter-quartile ranges are indicated by horizontal bars. The lion assay cut-off value (33 pg/ml) is shown as a dotted line on the y-axis (18). There was a statistically significant difference between the test results (p = 0.0001). High IFN-γ concentrations above the recombinant IFN-γ standard range (7.81 – 1000 pg/ml) for the assay were assigned the highest known standard concentration.

Using the African lion cut-off value (33pg/ml), the QFT Mabtech Cat IGRA correctly identified 3/6 *M. bovis* culture-confirmed leopards (**Figure 2**). Of the three TST-negative individuals with valid IGRA results, all three had little to no antigen-specific IFN-γ (**Figure 2**, **Supplementary Table S7**). However, there was no significant association observed between *M. bovis* infection status and QFT Mabtech Cat IGRA results (p = 0.24). Three out of seven leopards, with unknown infection status, were identified as IGRA positive (**Supplementary Table S7**). Two of the IGRA positive leopards also had GEA results, which were positive in the *CXCL9* GEA and showed upregulation of *CXCL10* (30 and 26.97-fold change; **Supplementary Table S4**).

**Figure 3:**
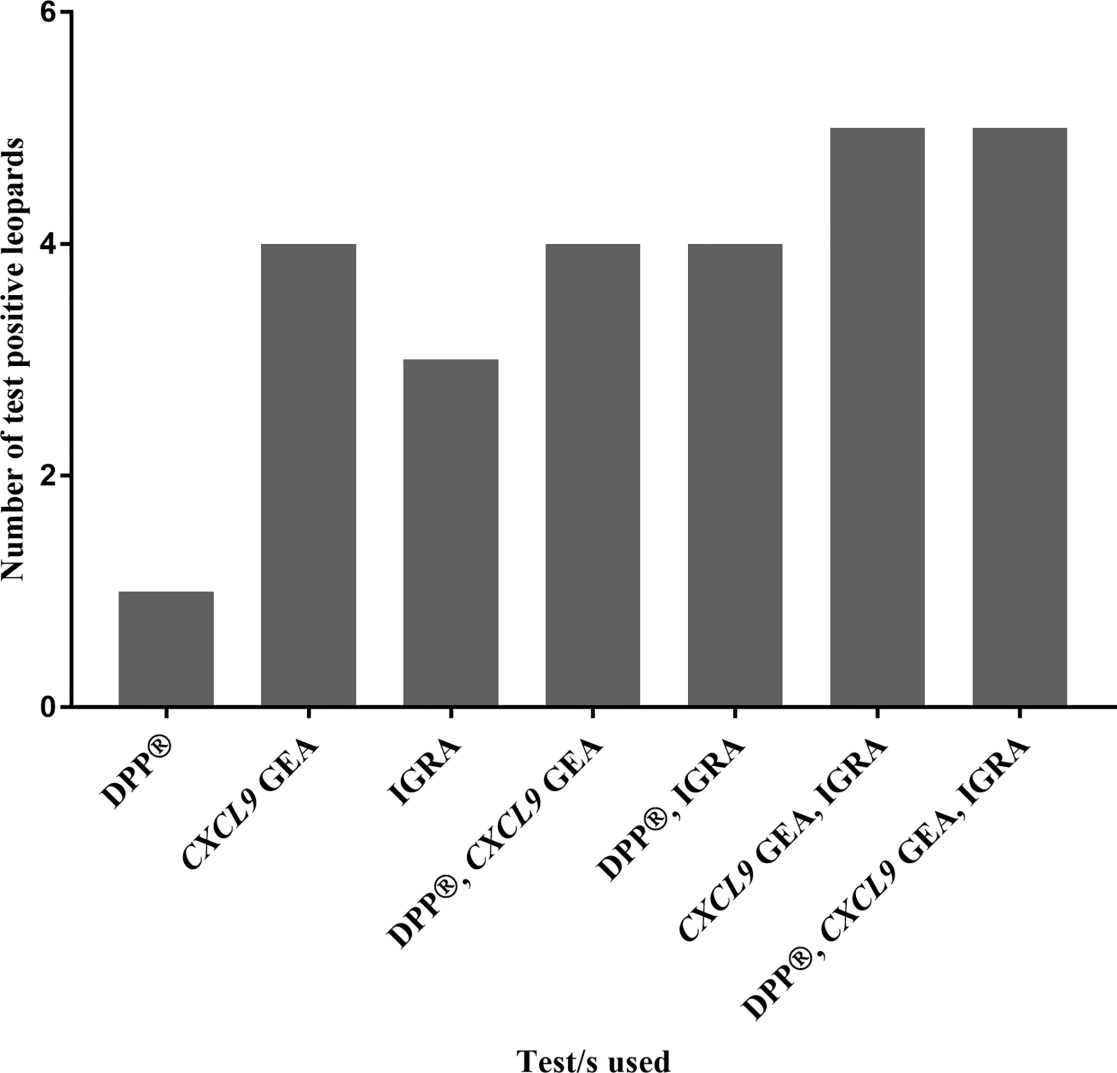
Numbers of test positive leopards, out of a cohort of *Mycobacterium bovis*-infected leopards (n = 6) confirmed by culture, are shown based on blood-based assay results (Dual Path Platform (DPP^®^) Vet TB Assay, QFT *CXCL9* gene expression assay, and QFT Mabtech Cat interferon gamma release assay) individually and in combination.

No single blood-based test nor parallel interpretation of combined tests was able to correctly identify all six culture positive leopards. However, when the a) *CXCL9* GEA and IGRA, and b) DPP^®^, IGRA, and *CXCL9* GEA were evaluated in parallel, 5/6 culture positive individuals were correctly identified (**Figure 3**).

## 4 Discussion

This study demonstrated that existing assays, previously evaluated for TB diagnosis in African lions (12, 16, 18, 31) and cheetahs (13), showed potential for detecting *M. bovis*-infected leopards. The GXU^®^ was able to detect MTBC DNA in all five culture positive leopards that were tested, providing rapid results in a significantly shorter time than mycobacterial culture. In addition, all *M. bovis* culture-confirmed leopards had positive results in the SICCT, supporting the use of the TST in leopards. Using blood-based assays, the QFT *CXCL9* GEA correctly identified 4/6 *M. bovis* culture-confirmed leopards using the African lion cut-off value. All except one *M. bovis* culture positive leopard also showed upregulation of antigen-specific *CXCL10* gene expression, although an assay cut-off value was unavailable. There was no antigen-specific cytokine upregulation observed in TST-negative leopards, suggesting that both GEAs were able to detect immune sensitization to *M. bovis*. In addition, the QFT domestic cat IGRA correctly identified 3/6 *M. bovis* culture-confirmed leopards using the African lion cut-off value, although only one infected leopard had detectable anti-mycobacterial antibodies. Parallel interpretation, using the QFT *CXCL9* GEA and IGRA, provided the most sensitive approach by identifying 5/6 *M. bovis* culture-confirmed leopards. Overall, findings in this pilot study support further investigations to determine leopard specific cut-off values and test performance of these assays in this species.

The GXU^®^ was 100% sensitive, providing a rapid ancillary diagnostic test for the detection of MTBC DNA (targeting IS*6110* and IS*1081*) from tissue homogenates, although it is unknown whether it will be sensitive when applied to respiratory samples, for antemortem diagnosis of leopards (21), or in leopards without TB-compatible lesions. Since vigorous decontamination of samples prior to mycobacterial culture can cause false negative results, GXU^®^ may enhance detection of MTBC in paucibacillary or non-viable MTBC samples, providing a valuable screening tool (32). However, the GXU^®^ cannot differentiate viable from non-viable mycobacteria nor distinguish between members of MTBC, which is important when investigating transmission.

The TST is the primary diagnostic tool used for the identification of *M. bovis*-infected domestic and wild animals (33). Although the TST has not been validated in most wild felid species, the findings in this study support its use in leopards. To account for possible cross-reactions with environmental mycobacteria, the SICCT was used since it takes into account responses to *M. avium* antigens (22, 23). Although studies in other species have reported that the SICCT has reduced sensitivity (34), all *M. bovis* culture-confirmed leopards (n=3) tested with SICCT in this study were positive. Interestingly, the TST in carnivores uses an increased volume of PPDs (0.2 ml), which is based on previous studies to optimize detection of a delayed-type hypersensitivity response (22). One of the limitations of the TST is the bias introduced through interpretation by different operators. Since TST is not validated in most wild species, the risk of misinterpretation remains.

Blood-based assays are logistically easier, and potentially less subjective to use in wildlife than the TST. Although assays to detect antimicrobial antibodies have been evaluated in wild carnivores, they appear to have suboptimal sensitivity unless disease is present (12, 13, 15). Therefore, it was expected that leopards with advanced disease might have detectable humoral response. One leopard (KNP-19/260), that showed extensive pulmonary pathological changes associated with TB, was positive for antibodies to mycobacterial antigen MPB83 using the DPP^®^ assay. However, the negative DPP^®^ results in the other *M. bovis* culture-confirmed leopards with disease could have been due to anergy associated with advanced disease or a humoral response to other mycobacterial antigens not included in the DPP^®^ assay. Typically, the humoral immune response in TB develops later in the course of infection and may take months to reach detectable levels (31, 35). Although the DPP^®^ assay does not appear to be sensitive for screening leopards, serological tests are useful since serum is easily obtained, tests are suitable for field use, results are rapid, and tests can be done with retrospective serum samples. Therefore, the DPP^®^ assay may be useful for screening selected leopards when disease is suspected.

Cell-mediated immune responses to TB are considered to be an early and more sensitive method to detect infection (36). Cytokine TB biomarker discovery has led to the development of antigen-specific GEAs in a number of species (11). Due to the taxonomic relatedness between lions and leopards, it was not surprising that the cytokine/chemokine primers for lions resulted in amplification of leopard target and reference genes (16, 37). Analyses showed the leopard sequences were a perfect match to those of the lion and supported further evaluation of lion *CXCL9* and *CXL10* GEAs in leopards. Results in leopards that exhibited poor mitogen and antigen-specific responses were considered invalid. However, some leopards had significant upregulation of antigen-specific cytokine genes expression, despite poor mitogen response. These results suggested that these individuals likely had *M. bovis* immune sensitization. Possible explanations for poor mitogen responses observed in leopards could be due to selection of mitogens (i.e., PHA and PWM not being optimal mitogens), sampling handling that affected viability, immunocompromise due to advanced disease, or suboptimal incubation time for blood stimulation (38–40). Therefore, further investigation of mitogen responses is required in leopards.

Both GEAs demonstrated the ability to detect antigen-specific upregulation of *CXCL9* and *CXCL10* in *M. bovis* culture-confirmed infected leopards. The upregulation of *CXCL9* has been reported as a sensitive diagnostic biomarker for *M. bovis* infection in African lions (16). However, in this study, both *CXCL9* and *CXCL10* showed an upregulation in some *M. bovis* culture-confirmed individuals, suggesting that these may be potential biomarkers for leopards. Additional studies should focus on optimizing these assays and determining leopard specific cut-off values, using a larger cohort with known infection status.

Although the cytokine GEAs showed promise, CRAs, especially IGRA, are more commonly used for screening humans and animals for TB (11, 36, 41). Even though all screened feline cytokine ELISA kits (TNF-α, IL-1β, and IFN-γ) were able to detect the cytokine of interest, the Mabtech Cat IFN-γ ELISA appeared to be the best for differentiating between *M. bovis* culture positive and TST-negative leopards. Due to the high homology (97-100%) between IFN-γ sequences from cheetahs, lions, and domestic cats (42), it was not surprising that the anti-cat IFN-γ antibodies were able to cross-react with native leopard, as well as cheetah and lion, IFN-γ in previous studies (13, 18).

The QFT Mabtech Cat IGRA correctly identified half of the *M. bovis* culture-confirmed leopards, using the African lion IGRA cut-off value, which suggests further investigations should determine if a leopard specific IGRA cut-off value would improve performance. It is also possible that lack of response was due to anergy, associated with advanced TB-related disease in two of the leopards (KNP-19/260 and KNP-22/853). However, these individuals exhibited valid mitogen responses. Therefore, it is crucial that immunoassay results are interpreted in conjunction with the history and clinical evaluation of leopards to avoid misclassification.

Even though both the QFT Mabtech Cat IGRA and *CXCL9* GEA correctly identified a proportion of TST-negative leopards (n=3; two had invalid IGRA responses) as negative (estimated 100% specificity), the calculated sensitivities of these assays were 50% and 67%, respectively. However, when these tests were interpreted in parallel, five of six *M. bovis* culture-confirmed leopards were correctly diagnosed.

Although the QFT Mabtech Cat IGRA, *CXCL9* and *CXCL10* GEAs demonstrated potential to identify *M. bovis* culture positive leopards and could be performed with a single blood sample, there were limitations to this study. While proportions of test positive individuals of *M. bovis* culture-confirmed and TST-negative leopards showed a trend, this requires further investigation to determine significance. In addition, there were no culture negative individuals to confirm absence of infection, some of the *M. bovis* culture-confirmed leopards had advanced disease (which could result in immunocompromise), and not every sample type was available for comparison in every individual. This preliminary study indicates that immunoassays may be useful for TB detection in leopards, but species-specific assay cut-off values should be determined in a larger study cohort of TB-endemic and TB-free leopard population to confirm the utility of this approach.

## 5 Conclusion

This pilot study suggested that blood-based assays used in parallel (QFT IGRA, QFT *CXCL9* GEA) show promise for detecting *M. bovis* culture positive leopards. However, future studies should validate use of the TST in leopards and the blood-based assays to improve performance and provide a single capture testing method. The incorporation of these diagnostic tools into routine screening of leopards and other wild felids for health assessment or as translocation candidates could improve TB detection and prevent spread of infection.

## Data availability statement

The datasets presented in this study can be found in online repositories. The names of the repository/repositories and accession number(s) can be found in the article/**Supplementary Material**.

## Ethics statement

The animal study was reviewed and approved by Stellenbosch University Animal Care and Use Research Ethics Committee.

## Author contributions

The work presented here was carried out in collaboration between all authors. RG, MM, and TK developed and designed the study. RG conducted experiments and analyzed the data. PB and L-MdK-L was involved with sample collection and clinical data. Original manuscript draft was prepared by RG and WG contributed to data analysis. MM, RW, PvH, and KL provided funding and feedback for the study. All authors contributed to the article and approved the submitted version.
