# Supplementary material for: “Spotting” Mycobacterium bovis infection in leopards (Panthera pardus) – novel application of diagnostic tools
Source: Front Immunol. 2023 Sep 1;14:1216262. doi: 10.3389/fimmu.2023.1216262 (PMC10505734; doi:10.3389/fimmu.2023.1216262)
Supplement: Supplementary file 1 [file DataSheet_1.pdf]

Supplementary Material

Supplementary Table 1. Tuberculin skin test (TST) results for free-ranging leopards.

Supplementary Table 2. Serological test results using the Dual Path Platform (DPP®) Vet TB Assay.

Supplementary Table 3. Real-time qPCR amplification efficiency for target and reference genes.

Supplementary Table 4. QFT *CXCL9* and *CXCL10* gene expression assay results of free-ranging leopards.

Supplementary Table 5. ELISA screening.

Supplementary Table 6. Selection of optimal ELISA using *M. bovis*-infected and uninfected leopards.

Supplementary Table 7. QFT Mabtech Cat IGRA results of free-ranging leopards.

Supplementary Table 1: Tuberculin skin test (TST) results for free-ranging leopards (*Panthera pardus*; n=8) sampled in Greater Kruger National Park, South Africa showing the initial cervical skin thickness measured in mm prior to PPD injection (0 hours) and after 72 hours. The single intradermal comparative cervical test (SICCT; positive result is an increase in skin thickness at the bovine PPD site ≥ 2 mm and the bovine PPD response greater than the avian PPD response) was used to classify the *Mycobacterium bovis* infection status of individuals. The single intradermal cervical test (SICT) was considered positive if the bovine PPD response was ≥ 2 mm.

Leopard Identification Number	Bovine PPD (mm)				Avian PPD (mm)			Bovine PPD – Avian PPD response (mm)	SICCT Result
	0 H	72 H	Bovine PPD response (72 H - 0 H)	SICT Result	0 H	72 H	Avian PPD response (72 H - 0 H)		
KNP- 19/260	6.5	10.8	4.3	pos	7.2	7.2	0	4.3	pos
KNP-19/07/01	8.9	19.0	10.1	pos	7.7	17.7	10.0	0.1	pos
KNP-19/279	5.7	24.0	18.3	pos	7.6	10.2	2.6	15.7	pos
KNP-11/121	n/a	n/a	n/a	neg*	n/a	n/a	n/a	n/a	neg*
KNP-11/236	n/a	n/a	n/a	neg*	n/a	n/a	n/a	n/a	neg*
KNP-16/155	6.4	6.9	0.5	neg	5.5	6.3	0.8	-0.3	neg
KNP-18/426	3.5	3.5	0	neg	3	4	1	-1	neg
KNP-20/58	7.4	7.6	0.2	neg	7.1	7.5	0.4	-0.2	neg

H: hours; mm: millimetres; PPD: purified protein derivative; SICCT: single intradermal comparative cervical test; SICT: single intradermal cervical test; pos: positive; neg: negative; n/a: not available; *: Test results taken from a veterinarian written report as numerical skin thickness measurements could not be retrieved due to computer malfunction.

Supplementary Table 2: Serological test results for free-ranging leopards (*Panthera pardus*; n=16) tested using the Dual Path Platform (DPP®) Vet TB Assay. A reflective light unit (RLU) measurement ≥ 5 was considered test positive.

Leopard Identification Number	Test line 1 MPB83 (RLU)	Test line 2 CFP10/ESAT-6 (RLU)	Test Result
KNP- 19/260*	49.9	0	pos
KNP-19/07/01*	2.9	0	neg
KNP-19/279*	2.8	0	neg
KNP-18/660*	0	0	neg
KNP-22/728*	2.8	0	neg
KNP-22/853*	3.9	0	neg
KNP-16/155‡	3.0	0	neg
KNP-18/426‡	0	0	neg
KNP-20/58‡	0	0	neg
KNP-17/682	0	0	neg
KNP-17/752	0	0	neg
KNP-18/35	0	0	neg
KNP-18/234	0	0	neg
KNP-18/526	2.7	0	neg
KNP-18/425	0	0	neg
KNP-22/576	3.1	0	neg

MBP83: mycobacterial protein bovis 83; ESAT-6: early secretory antigenic target 6 kDa; CFP-10: culture filtrate protein 10 kDa; RLU: reflective light units; pos: positive; neg: negative; **Mycobacterium bovis*-infected leopards; ‡ tuberculin skin test negative leopards.

Supplementary Table 3: Real-time qPCR amplification efficiency for target and reference genes, showing the coefficient of correlation (R^2) and slope of the standard curve.

**Gene**	**R^2**	**Slope**	**Efficiency (%)**
CXCL9	1.00	-3.35	99
CXCL10	1.00	-3.28	102
IFN-γ	0.98	-3.04	113
YWHAZ	1.00	-3.41	96
GAPDH	1.00	-3.27	102
B2M	1.00	-3.26	103
TBP	1.00	-2.71	134

CXCL9: C-X-C motif ligand 9; *CXCL10*: C-X-C motif chemokine ligand 10; *IFN-γ*: interferon-gamma; *YWHAZ*: tyrosine 3-monooxygenase/tryptophan 5-monooxygenase activation protein, zeta polypeptide; *GAPDH*: glyceraldehyde-3-phosphate dehydrogenase; *B2M*: β-2-microglobulin; *TBP*: TATA box-binding protein.

Supplementary Table 4: QuantiFERON®-TB Gold Plus (QFT) C-X-C motif ligand 9 (*CXCL9*) and C-X-C motif chemokine ligand 10 (*CXCL10*) gene expression assay (GEA) results of free-ranging leopards (*Panthera pardus*; n=15) sampled in Greater Kruger National Park, South Africa. A 5-fold change ($2^{-\Delta\Delta cq} \geq 5$) previously calculated for African lions (*Panthera leo*) (16), was considered test positive for *CXCL9*. In the absence of an existing *CXCL10* cut-off value, the GEA test results for *CXCL10* were unknown.

Leopard Identification Number	QFT *CXCL9* GEA			QFT *CXCL10* GEA		
	**Mitogen specific fold change ($2^{-\Delta\Delta cq}$)**	**TB2 antigen specific fold change ($2^{-\Delta\Delta cq}$)**	**Test Result lion cut-off (5-fold change)**	**Mitogen specific fold change ($2^{-\Delta\Delta cq}$)**	**TB2 antigen specific fold change ($2^{-\Delta\Delta cq}$)**	**Test Result**
KNP-19/260*	0.09	13.03	pos	0.04	16.91	unknown
KNP-19/07/01*	0.03	1.62	neg	0.08	2.23	unknown
KNP-19/279*	0.13	13.93	pos	0.74	7.29	unknown
KNP-18/660*	9.51	1223.39	pos	2.29	176.88	unknown
KNP-22/728*	104.33	40.64	pos	49.41	17.59	unknown
KNP-22/853*	6.14	4.42	neg	17.92	53.20	unknown
KNP-16/155‡	1.05	0.95	neg	2.97	0.12	unknown
KNP-18/426‡	3.96	1.49	neg	1.51	1.57	unknown
KNP-20/58‡	371.36	1.83	neg	100.89	1.62	unknown
KNP-14/228	78.25	7.82	pos	108.63	4.44	unknown
KNP-17/752	134.99	146.02	pos	12.10	26.97	unknown
KNP-18/35	1.64	0.37	neg	0.74	1.42	unknown
KNP-18/234	4.32	1.78	neg	3.87	2.36	unknown
KNP-18/526	66.87	3.62	neg	20.35	0.98	unknown
KNP-18/425	1.95	17.51	pos	17.51	30.00	unknown

**Mycobacterium bovis*-infected leopards; ‡ tuberculin skin test negative leopards; pos: positive; neg: negative.

Supplementary Table 5: Cytokine concentrations (pg/ml) measured using commercial feline cytokine ELISA kits in serially diluted plasma from separately pooled unstimulated (nil) and mitogen stimulated leopard whole blood samples. Concentrations were based on extrapolation from known domestic cat recombinant cytokine standard curves.

Cytokine ELISA	Recombinant Cytokine Standard range (pg/ml)	Stimulation	Sample Concentration (pg/ml)				SD	CV (%)
			Sample dilution			Mean		
			1:2	1:4	1:8			
R&D Feline IL-1β	31.3 - 2000	QFT nil	71	0	0	23.67	40.99	173.3
		QFT mitogen	> 2000	> 2000	> 2000	NC	NC	NC
R&D Feline TNF-α	15.6 - 1000	QFT nil	429	450	384	421	33.72	8.0
		QFT mitogen	> 1000	> 1000	> 1000	NC	NC	NC
Mabtech Cat IFN-γ	7.81 - 1000	QFT nil	102	113	150	121.67	25.15	20.7
		QFT mitogen	> 1000	> 1000	> 1000	NC	NC	NC

QFT: QuantiFERON®-TB Gold Plus; IL-1β: interleukin-1 beta; TNF-α: tumour necrosis factor alpha; IFN-γ: interferon gamma; SD: standard deviation; CV: coefficient of variation; Nil: unstimulated negative control; Mitogen: mitogen; stimulated positive control; NC: not calculated.

Supplementary Table 6: QuantiFERON®-TB Gold Plus (QFT) cytokine release assay results for three TB-confirmed and one TST-negative leopard. Antigen-specific cytokine concentrations (QFT TB2 – QFT nil) were considered valid when cytokine concentrations in QFT mitogen stimulated blood samples were substantially higher than in QFT nil samples ($p < 0.05$). Calculated cytokine concentrations above the upper limit of the standard curves were set at the known upper limit of the standard range.

**Leopard Identification Number**	**Cytokine Release Assay Results**					
	R&D Feline IL-1β		R&D Feline TNF-α		Mabtech Cat IFN-γ	
	Validation (QFT mitogen-QFT nil) (pg/ml)	Result (QFT TB2-QFT nil) (pg/ml)	Validation (QFT mitogen-QFT nil) (pg/ml)	Result (QFT TB2-QFT nil) (pg/ml)	Validation (QFT mitogen-QFT nil) (pg/ml)	Result (QFT TB2-QFT nil) (pg/ml)
KNP-19/260*	> 2000	93	128	0	56	0
KNP-19/07/01*	> 2000	0	329	0	> 1000	> 1000
KNP-19/279*	> 2000	414	477	103	> 1000	> 1000
KNP-18/426‡	1 553	0	12	0	53	12

QFT: QuantiFERON®-TB Gold Plus; IL-1β: interleukin-1 beta; TNF-α: tumour necrosis factor alpha; IFN-γ: interferon gamma; Nil: unstimulated negative control; Mitogen: mitogen stimulated; TB2: specific antigenic peptides; **Mycobacterium bovis*-infected leopards; ‡ tuberculin skin test negative leopard.

Supplementary Table 7: QuantiFERON®-TB Gold Plus (QFT) Mabtech Cat interferon gamma release assay (IGRA) results of free-ranging leopards (*Panthera pardus*; n=18) sampled in Greater Kruger National Park, South Africa. Results were considered valid when interferon gamma (IFN-γ) concentrations in QFT mitogen stimulated blood samples were significantly greater than in QFT nil samples ($p < 0.05$). An antigen-specific IFN-γ concentration ≥ 33 pg/ml was classified as a positive result for the IGRA, based on the previously calculated cut-off value for African lions (*Panthera leo*) (18). Calculated IFN-γ concentrations above the known upper limit (1000 pg/ml) of the assay were assigned a value > 1000 pg/ml.

**Leopard Identification Number**	**QFT Mabtech Cat IGRA RESULTS**		
	**Validation (mitogen-nil) (pg/ml)**	**Result (TB2 - nil) (pg/ml)**	**Test Result lion cut-off (33 pg/ml)**
KNP-19/260*	56	0	neg
KNP-19/07/01*	>1000	>1000	pos
KNP-19/279*	>1000	>1000	pos
KNP-18/660*	282	>1000	pos
KNP-22/728*	>1000	15	neg
KNP-22/853*	661	0	neg
KNP-11/121‡	0	0	invalid
KNP-11/236‡	>1000	0	neg
KNP-16/155‡	0	0	invalid
KNP-18/426‡	53	12	neg
KNP-20/58‡	>1000	0	neg
KNP-14/228	>1000	18	neg
KNP-17/752	366	44	pos
KNP-18/35	185	0	neg
KNP-18/234	54	0	neg
KNP-18/526	>1000	0	neg
KNP-18/425	>1000	328	pos
KNP-22/576	593	146	pos

IGRA: interferon gamma release assay; QFT: QuantiFERON®-TB Gold Plus; Nil: unstimulated negative control; Mitogen: mitogen stimulated; TB2: specific antigen peptides; **Mycobacterium bovis*-infected leopards; ‡ tuberculin skin test negative leopards; pos: positive; neg: negative.
